# On the influence of cannabinoids on cell morphology and motility of glioblastoma cells

**DOI:** 10.1371/journal.pone.0212037

**Published:** 2019-02-12

**Authors:** Tim Hohmann, Kerstin Feese, Chalid Ghadban, Faramarz Dehghani, Urszula Grabiec

**Affiliations:** Institute of Anatomy and Cell Biology, Martin Luther University Halle-Wittenberg, Halle (Saale), Germany; Duke University School of Medicine, UNITED STATES

## Abstract

The mechanisms behind the anti-tumoral effects of cannabinoids by impacting the migratory activity of tumor cells are only partially understood. Previous studies demonstrated that cannabinoids altered the organization of the actin cytoskeleton in various cell types. As actin is one of the main contributors to cell motility and is postulated to be linked to tumor invasion, we tested the following hypothesizes: 1) Can cannabinoids alter cell motility in a cannabinoid receptor dependent manner? 2) Are these alterations associated with reorganizations in the actin cytoskeleton? 3) If so, what are the underlying molecular mechanisms? Three different glioblastoma cell lines were treated with specific cannabinoid receptor 1 and 2 agonists and antagonists. Afterwards, we measured changes in cell motility using live cell imaging and alterations of the actin structure in fixed cells. Additionally, the protein amount of phosphorylated p44/42 mitogen-activated protein kinase (MAPK), focal adhesion kinases (FAK) and phosphorylated FAK (pFAK) over time were measured. Cannabinoids induced changes in cell motility, morphology and actin organization in a receptor and cell line dependent manner. No significant changes were observed in the analyzed signaling molecules. Cannabinoids can principally induce changes in the actin cytoskeleton and motility of glioblastoma cell lines. Additionally, single cell motility of glioblastoma is independent of their morphology. Furthermore, the observed effects seem to be independent of p44/42 MAPK and pFAK pathways.

## Introduction

Malignant tumors still belong to the most common causes of death worldwide with an increasing tendency [1,2]. One of the most lethal tumor types is the glioblastoma multiforme (GBM) that is highly resistant to the standard therapy [3–8]. This resistance against standard therapy arises in part by the diffuse infiltration pattern into the surrounding brain, making a complete resection nearly impossible [9–12]. The invasion of tumor cells into adjacent tissue is generally controlled by a multitude of processes demanding structural adaption of single tumor cells. This includes an initial reduction in cell adhesion, a degradation of the surrounding extracellular matrix, and a subsequent (directed) movement away from the main tumor. In case of glioblastoma, single cells detach from the main tumor mass and produce finger-like protrusions, forming new attachments at the cell front while releasing the rear [13]. The biological process regulating cell motility is mainly governed by the cytoskeleton, including dynamic remodeling of the actin and microtubule network [14–16].

One potential modulator of cell motility might be the cannabinoid system. In different cell types the modulation of the activity of cannabinoid receptors (CB) CB_1_ and CB_2_ resulted in changes of cell motility as well [17–21]. In our previous study we examined amongst others the effects of the two specific CB_1_ and CB_2_ agonists namely ACEA and JWH133 on motility and invasion properties of glioblastoma cell lines and observed cell line dependent effects [21]. Studies in other systems showed e.g. a CB_2_ dependent inhibition of migration in bladder cancer cell lines [17], while another work reported about a CB_1_ dependent inhibition of breast cancer cell migration [18]. Both studies found an association with focal adhesion kinases (FAK) signaling [17,18]. In another investigation the observed CB_1_ dependent inhibition of motility in prostate carcinoma cells was caused by an inhibition of the small GTPase RhoA, with an accompanying increase in Cdc42 and Rac1 activity [19]. Beyond these CB specific effects, one group reported a cannabinoid receptor independent effect of the CB_1_/CB_2_ antagonist cannabidiol that led to an inhibition of glioma cell migration [22]. Additionally, Δ9-tetrahydrocannabinol (THC) was found to inhibit the epithelial growth factor-induced cell migration of lung cancer cells via inhibition of ERK1/2 and AKT [23].

Current research indicates the presence of the two well characterized cannabinoid receptors CB_1_ and CB_2_ in human glioma and glioblastoma [24,25]. Cannabinoids have previously shown to exert anti-tumoral effects in vitro in a multitude of tumor types, leading to apoptosis, cell cycle arrest and a reorganization of cytoskeletal components [26–36]. These anti-tumoral effects were frequently associated with a reduced phosphorylation of p44/42 MAPK and changes in FAK phosphorylation, both being involved in control of cell motility and in case of FAK also with cell-matrix adhesion [18,23,37–41].

Thus, in this study we examined the effects of specifically modulating both cannabinoid receptors CB_1_ and CB_2_ on cell motility, cell morphology, actin cytoskeleton, p44/42 MAPK and FAK phosphorylation in glioblastoma cells.

## Materials and methods

### Cell culture

U87 and LN229 cells were purchased from the American Type Culture Collection (Manassas, VA, USA; U87: ATCC HTB-14; LN229: ATCC CRL-2611) and U138 cells were obtained from Cell Lines Service (Cell Lines Service, 300363). All cell lines were cultured as described previously [21]. 24 hours prior to the start of any experiment the culture medium was changed. Cannabinoid receptor agonists (both Tocris, Bristol, UK; CB_1_ agonist ACEA; dissolved in ethanol; 1319 or CB_2_ agonist JWH-133 dissolved in DMSO; 1343) were added with a concentration of 10 μM and cannabinoid receptor inverse agonists (both Tocris; CB_1_ antagonist/inverse agonist AM281; dissolved in DMSO; 1115 or CB_2_ antagonist/inverse agonist AM630 dissolved in DMSO; 1120) were used at a concentration of 1 μM. If the cells were treated with both agonists and inverse agonists, the inverse agonist was applied 15 min before the agonist.

### Time lapse microscopy

For time lapse microscopy 1,000 cells were seeded in a 12-well plate 24 hours prior to the start of experiments. Images were taken with a microscope (Leica DMi8, Leica, Wetzlar, Germany) equipped with temperature (37°C) and CO_2_ regulation (5% (v/v)). The experiments were conducted as described previously [21]. Briefly, using the sobel operator and subsequent morphological operations were used to detect the cells outline. Thereby we determine the parameters contact area, directionality, mean speed, optical homogeneity, apparent intensity [42] and the circularity or index of ramification [43]. Analysis was performed using the same self-written MATLAB scripts (The Mathworks, Nattick, MA, USA) as described previously [21].

The directionality is defined as the ratio of the total distance traveled by the cell divided by the net distance from the starting point. Consequently, a value of one describes a straight line, while higher values indicate a less direct path. The circularity or index of ramification is a morphological measure describing the difference between the geometric pattern of the cell and a circle. It is calculated as the ratio between the area of a circle with a circumference that is equal to the outline of the cell and the area of the cell. For a cell with an area *A* and a circumference *U* the circularity *c* is defined as: c = 4*π*A/U^2^. The optical homogeneity was defined as the variance of the brightness of each pixel inside the cell and thus high values correspond to heterogeneous cells while low values correspond to uniform cells [42]. Similarly, the apparent intensity/brightness was defined as the mean value of the brightness of each pixel inside a cell divided by the mean brightness of pixels outside the cell [42]. As phase contrast microscopy was used the apparent brightness is correlated to the optical density of the cell.

### Immunofluorescence and immunhistochemical staining

24 hours after cannabinoid treatment 50,000 cells were placed on glass cover slips coated with poly-L-lysin (Carl Roth, Karlsruhe Germany) and incubated for another 24 h till the fixation with 4% paraformaldehyde for 10 min. For actin labelling we used a phalloidin-488 staining. Cells were washed twice for 10 min in 0.1% PBS/Triton solution, then with PBS and blocked with 1% bovine serum albumin. An incubation step with phalloidin-488 (2.5 μl/100 μl BSA solution, Thermo Fisher Scientific, Waltham, MA, USA, A12379) was performed for 20 min. For the visualisation of the nucleus 4’,6-Diamin-2-phenylindol (DAPI, 1:10000, Sigma Aldrich, Saint Louis, MI, USA, D9542) was used. The stained cells were washed with both PBS and distilled water and covered with DAKO mounting medium (DAKO, Santa Clara, CA, USA).

Images of phalloidin-stained cells were acquired with a 63× objective using a confocal laser scanning microscope. The following excitation wavelengths were used: 405 nm for DAPI and 488 nm for phalloidin. Emission was detected in the range of Δλ = 400–480 nm (DAPI) and Δλ = 500–650 nm (phalloidin).

For evaluation of cytoskeletal alterations we used an approach described elsewhere that is based on the image coherency [44]. This approach assumes that the overall structure can be understood as the sum over all local structures of actin fibers inside the cell. Thereby, the structure density can be obtained as the structuredness normalized to the cell area. The images were analyzed using a self-written MATLAB (The MathWorks, Natick, USA) script.

### Western blotting

The western blot analysis was performed as described before [21]. The cells were collected 0 min, 5 min, 10 min, 30 min, 2 h, 12 h, 24 h and 72 h after cannabinoid treatment in 75 μl sample buffer. 10 μg of the sample were loaded on the electrophoresis gel. The analyses were performed as described earlier [21,45]. The list of used antibodies is attached in Table 1. The imaging and evaluation of blots was done using the Fusion FX7 (PeqLab).

**Table 1:** Used antibodies and their dilution.

Antibody	Dilution	Company	Catalog number	Antibody ID	Clonality	Target Antigen
phospho-p44/42 MAPK (Erk1/2) (Thr202/Tyr204)	1:4000	Cell Signaling (Cambridge, UK)	9101	AB_331646	Polyclonal	P44/42 MAPK, phospho Thr202/Tyr204
t-p44/42 MAPK (Erk1/2)	1:4000	Cell Signaling	9102	AB_330744	Polyclonal	P44/42 MAPK
pFAK (Tyr925)	1:1000	Cell Signaling	3284S	AB_2253227	Polyclonal	FAK, phospho (Tyr925)
FAK	1:1000	Cell Signaling	3285S	AB_10694068	Polyclonal	FAK
GAPDH	1:1000	Cell Signaling	2118L	AB_561053	Monoclonal	GAPDH
goat anti-rabbit IgG, HRP conjugated	1:20000	Vector laboratories (Burlingame, CA, USA)	PI-1000	AB_2336198	unknown	Rabbit IgG

### Statistics

Statistics was performed using the two-sided Mann-Whitney-Wilcoxon test or Kruskal-Wallis test. Significance was chosen for p<0.05.

## Results

### Cannabinoids influence motile properties of single glioblastoma cells

**Fig 1:**
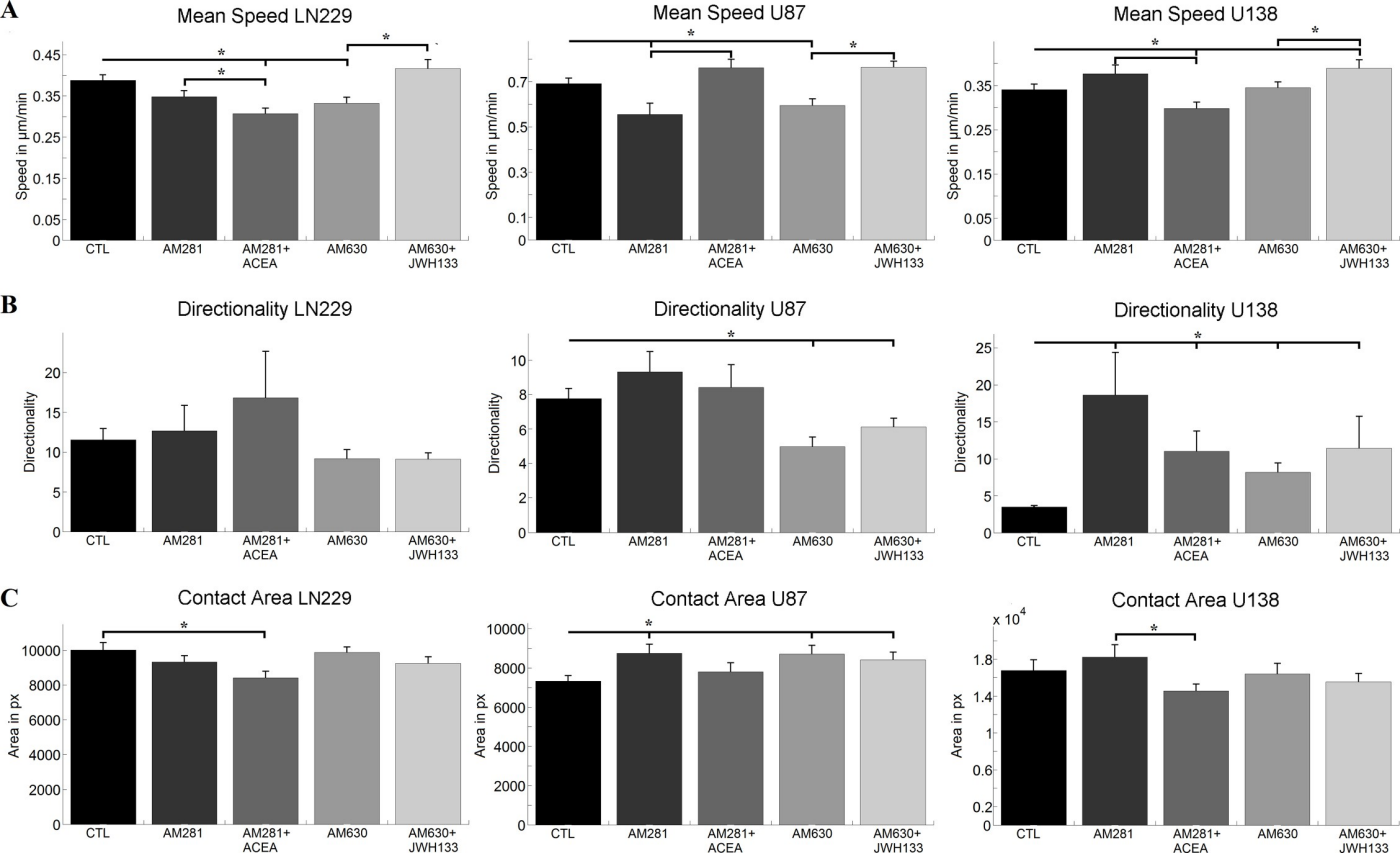
Impact of cannabinoids on motility related properties of single cells. A) shows the mean speed and standard error of the mean (sem) of single LN229, U87 and U138 cells, when treated with cannabinoid receptor inverse agonists. B) and C) depict the directionality and contact area of the same cell lines and treatments together with the sem. For all parameters cell line specific changes that have no apparent receptor specificity can be observed. Statistics was performed using a Kruskal-Wallis test and significance was chosen for p<0.05. The asterisk denotes significant results regarding the respective measurement indicated with the bar.

The three used cell lines differed in their basal motile properties as well as in their sizes. U87 (v = 0.69 μm/min) cells appeared to be fastest and U138 (v = 0.34 μm/min) cells were only slightly slower than LN229 (v = 0.39 μm/min; Fig 1A). Regarding the movement pattern, LN229 moved in the least straight manner (d = 11.6), followed by U87 (d = 7.8) and U138 cells (d = 3.5; Fig 1B). In terms of size, U138 cells had the by far largest contact area to the substrate (A = 16766 px), being roughly 1.7 and 2.3 times larger than LN229 (A = 10015 px) or U87 (A = 7335 px) cells, respectively (Fig 1C).

When measuring the mean speed we observed a decrease in cell speed after application of the CB1 inverse agonist AM281 for U87 cells only, while it had no effect on cell speed of both U138 and LN229 cells. A co-application of AM281 together with the CB1 agonist ACEA led to a decrease in cell speed of LN229 and U138 cells, but did not alter the motility of U87 cells relative to the control. Treatment of LN229 and U87 cells with the CB2 inverse agonist AM630 led to an decrease in cell motility but had no effect on U138 cell speed. AM630 together with the CB2 agonist JWH133 did not alter cell speed relative to the control conditions in LN229 and U87 cells but led to an increase in U138 cells (Fig 1A).

While the cannabinoid treatments had no effect on the movement pattern of LN229 cells, we observed a decrease in directionality (straighter cell path) after the application of the CB2 inverse agonist, as well as for the co-application with the CB2 agonist in U87 cells. In contrast, all cannabinoid treatments led to an increase in directionality (less straight movement) in U138 cells (Fig 1B).

The measurements of the contact area of LN229 cells resulted in a decreased contact area, when compared to the control, for the combination of the CB1 inverse agonist and the CB1 agonist only. In contrast, the contact area of U87 cells increased when treated with the CB1 inverse agonist, the CB2 inverse agonist and the combination of the CB2 inverse agonist and the CB2 agonist. For U138 cells a significant difference was observed between the treatment with the inverse CB1 agonist and the combination of CB1 agonist and inverse agonist (Fig 1C).

The sample size for each cell type and treatment was at least 40. The exact measurement values and sample sizes are shown in supplemental S1–S3 Tables.

### Cannabinoids influence the morphology of single glioblastoma cells

**Fig 2:**
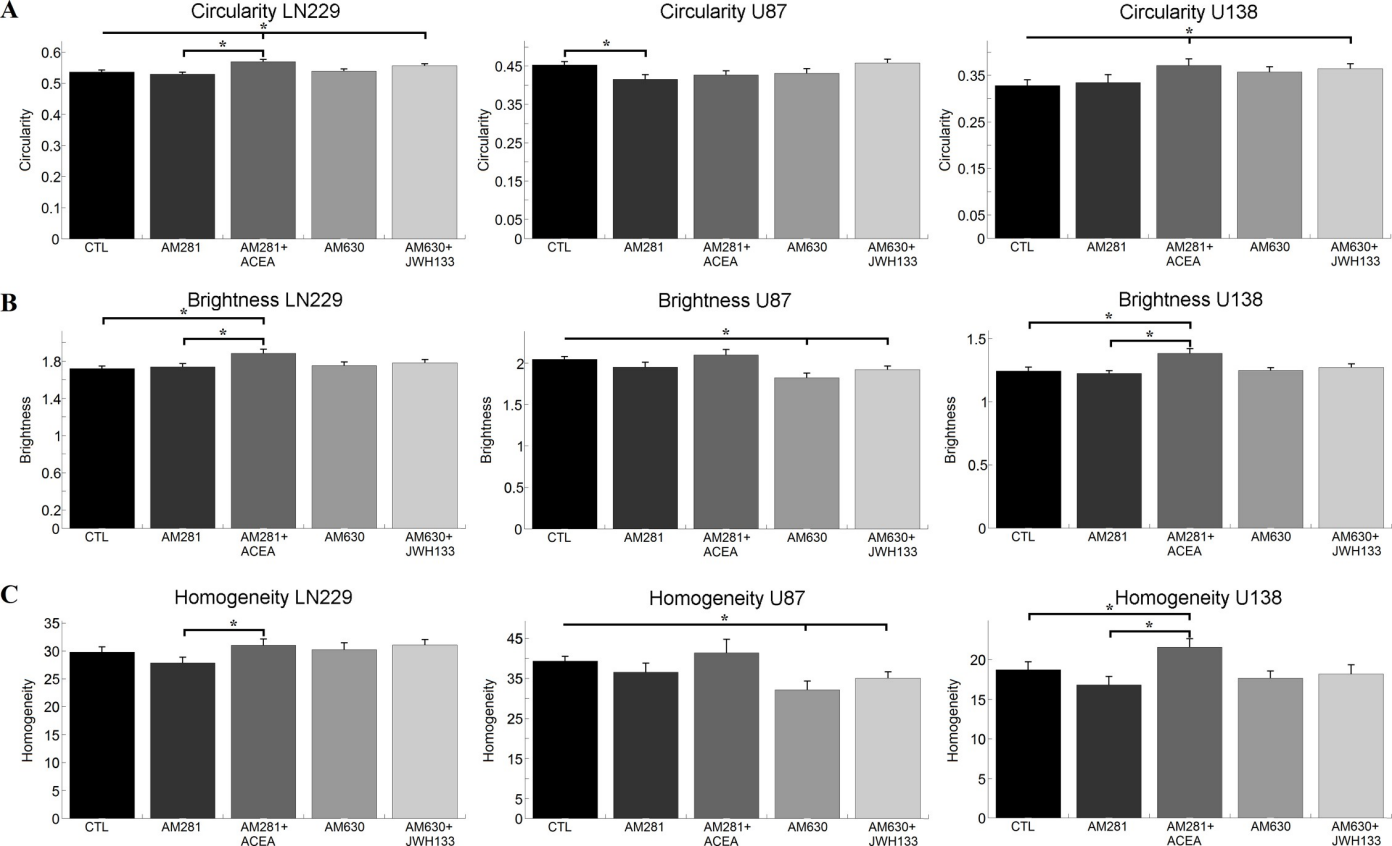
Impact of cannabinoids on morphological parameters of single cells. A) shows the mean circularity and sem of single LN229, U87 and U138 cells, when treated with cannabinoid receptor inverse agonists. B) and C) depict the apparent brightness and homogeneity of the same cell lines and treatments together with the sem. For all parameters cell line specific changes that have no apparent receptor specificity can be observed. Statistics was performed using a Kruskal-Wallis test and significance was chosen for p<0.05. The asterisk denotes significant results regarding the respective measurement indicated with the bar.

Despite motile properties of single cells we evaluated morphological properties, including the circularity, homogeneity and brightness of each cell. Thereby, distinct differences have been spotted between the cell lines: LN229 cells appeared to be most circular (c = 0.536), while U138 (c = 0.328) cells deviated strongly from a circular shape. Regarding the brightness of each cell, U87 were brightest (I = 2.04) and U138 (I = 1.24) cells were only slightly brighter than the background. Similarly, U87 (h = 39.3) cells were the least homogeneous, while U138 (h = 18.9) cells were most homogeneous on average (Fig 2).

Calculating the circularity of LN229 and U138 cells after cannabinoid treatments, significant effects were only found after the co-application of the CB1/CB2 inverse agonist and agonist together, resulting in a more circular cell shape. For U87 cells only the CB1 inverse agonist AM281 resulted in less circular shapes (Fig 2A).

The analysis of the brightness depicted that the co-administration of AM281 and ACEA led to an increased brightness in both LN229 and U138 cells when compared to the control and the inverse agonist alone. In contrast, both the CB2 inverse agonist AM630 and AM630 together with the CB2 agonist JWH 133 resulted in less bright U87 cells (Fig 2B).

The study of the homogeneity of the cells after cannabinoid treatment showed a significant difference for LN229 cells between AM281 and AM281+ACEA only. For U87 cells a reduced heterogeneity was observed after AM630 and AM630+JWH 133 treatment. U138 cells got more heterogeneous, when treated with AM281+ACEA compared to the control measurement and the treatment with the CB1 inverse agonist alone (Fig 2C).

The sample size for each cell type and treatment was at least 40. The exact measurement values and sample sizes are shown in supplemental S4–S6 Tables.

### Correlation of single cell parameters

**Fig 3:**
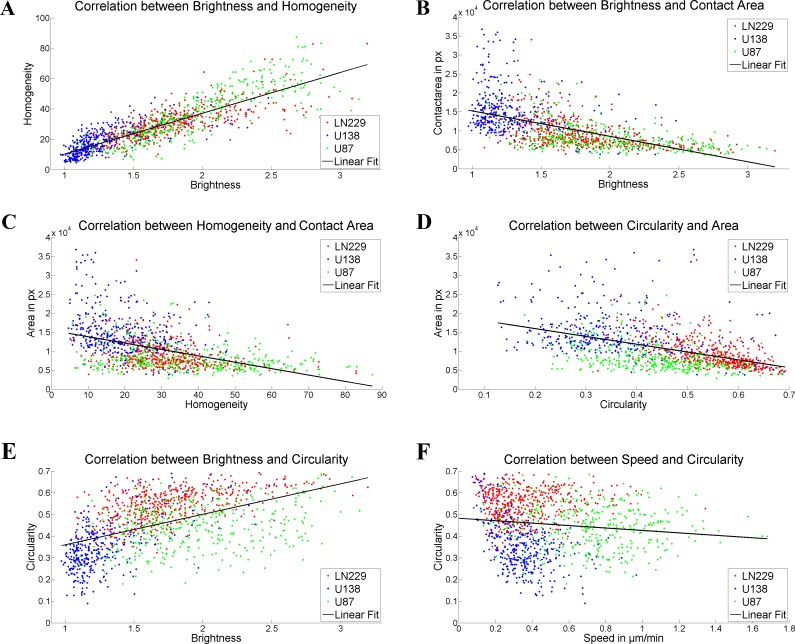
Correlation of single cell parameters. A) shows the correlation between apparent brightness and homogeneity of the three cell lines, with an correlation coefficient of r = 0.848 [0.831;0.863]. B), C), D), E) and F) illustrate the correlation between the single cell parameters brightness and contact area, homogeneity and contact area, circularity and contact area, brightness and circularity as well as cell speed and circularity, with correlations coefficients of r = -0.576 [-0.612;-0.536], r = -0.456 [-0.500;-0.410], r = -0.480 [-0.522;-0.435], r = 0.516 [0.473;0.556] and with r = -0.122 [-0.177; 0.066], respectively. Red dots correspond to LN229 cells, blue ones to U138 cells and green dots to U87 cells. The black line corresponds to the respective linear fit.

To evaluate whether certain parameters are independent of each other or allow a prediction on the evaluation of the respective other ones Pearson correlation coefficients were calculated between all single cell parameters. We thereby disregarded all correlations that were found for the three cell populations pooled together but not for each individual cell line. With this analysis a strong correlation was found between the morphological parameters brightness and homogeneity (r = 0.848 [0.831;0.863]) (Fig 3A) and consequently between the morphological parameters brightness/homogeneity and the contact area with r = -0.576 [-0.612;-0.536] or r = -0.456 [-0.500;-0.410] (Fig 3B and 3C), respectively. Additionally, the circularity was correlated with the contact area and brightness with r = -0.480 [-0.522;-0.435] and r = 0.516 [0.473;0.556] (Fig 3D and 3E). Notably, no significant correlation between circularity and cell speed was found: r = -0.122 [-0.177; 0.066] (Fig 3F). Notably, the cannabinoid treatment did not impact the observed correlations.

### Influence of cannabinoids on the actin structure of single glioblastoma cells

**Fig 4:**
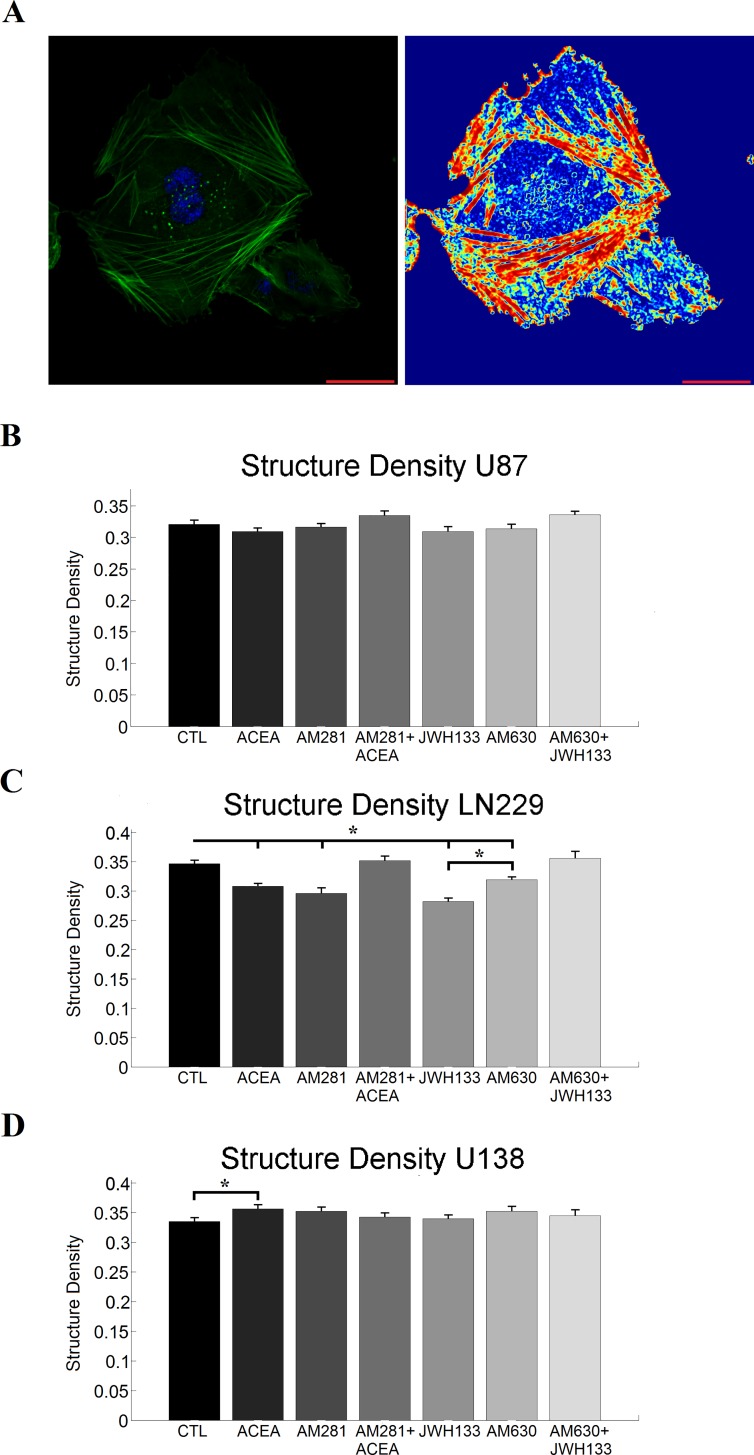
Actin structure measurements. A) depicts the phalloidin (green) and DAPI (blue) staining of LN229 control cells on the left and the respective structure image of the actin cytoskeleton as a heat map on the right. A correspondence of highly structured regions in the actin staining with the respective structure image is visible. Furthermore, unstructured, homogeneous regions do not contribute to the structure image, as for example in the center of the image. The scaling corresponds to 20 μm. B) shows the quantification of the density of actin structures for U87 cells treated with cannabinoid receptor agonists and inverse agonists as mean value with the sem. C) and D) illustrates the density of actin structures in LN229 and U138 cells after cannabinoid treatment. Various effects that show no apparent receptor specificity were observed. Statistics was performed using a Kruskal-Wallis test and significance was chosen for p<0.05. The asterisk denotes significant results regarding the respective measurement indicated with the bar.

The analysis of the actin staining revealed the expected structure and dense actin network of the glioblastoma cells (Fig 4A). We observed a clearly visible peripheral actin structure and dense arrays of mostly parallel stress fibers. Protrusive actin appeared as dense clusters at cell edges, while punctuate actin appeared as bright dots inside the cytoplasm. For U87 cells neither CB agonists, antagonists nor the combination caused a significant change in actin structure density (Fig 4B). For LN229 cells we could observe a decreased density of actin structures after treatment with CB agonists or antagonists that were abolished when the agonists and antagonists were administered together (Fig 4C). In contrast, the CB1 agonist ACEA led to an increase in structure density in U138 cells, while the remaining treatments had no significant effect (Fig 4D). The exact measurement values are shown in supplemental S7 Table.

### Influence of cannabinoids on FAK and p44/42 MAPK expression of glioblastoma cells

**Fig 5:**
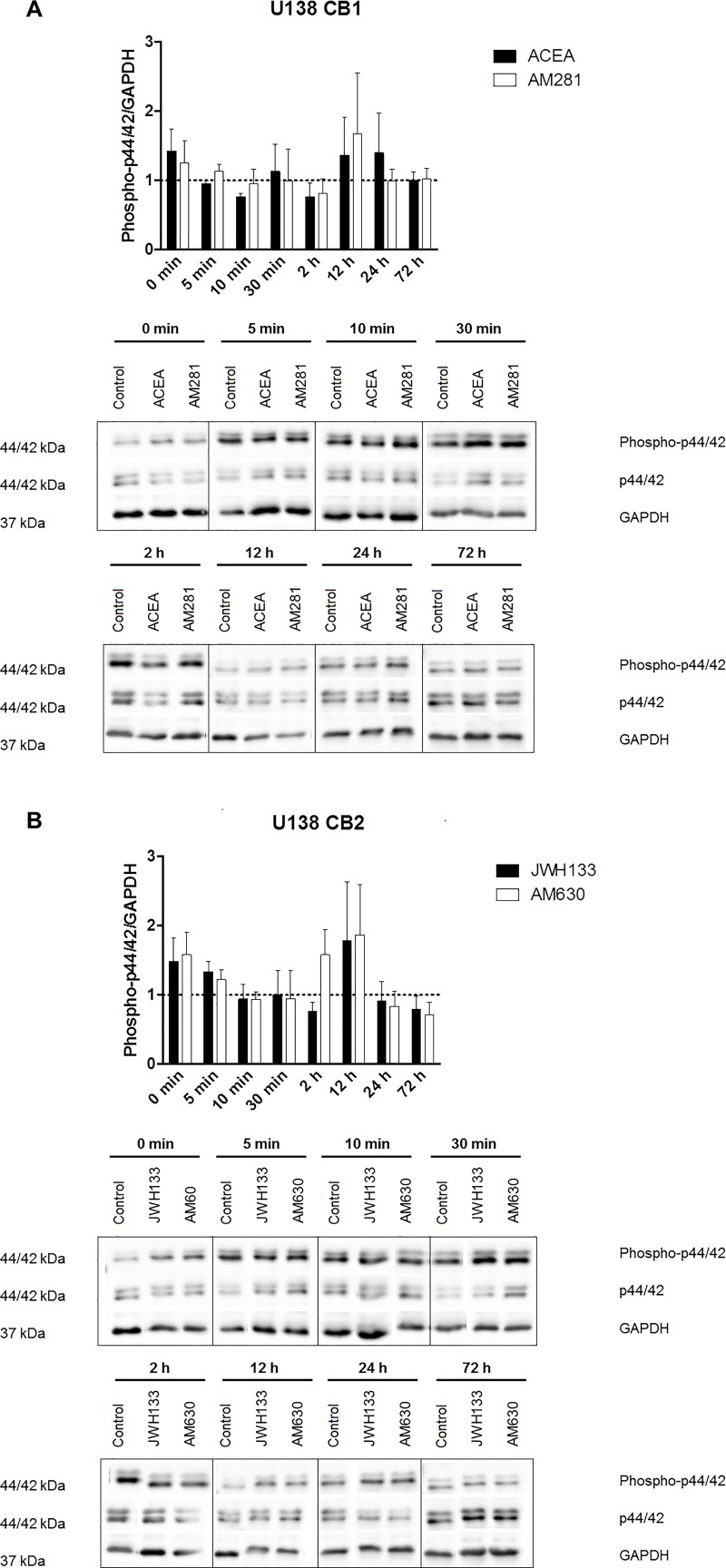
P44/42 MAPK phosphorylation of U138 cells after cannabinoid treatment. A) depicts the phosphorylation of p44/42 MAPK of U138 cells after CB1 agonist and inverse agonist treatment. B) shows the phosphorylation of p44/42 MAPK of U138 cells after CB2 agonist and inverse agonist treatment. All values depict the mean of the measurements together with the sem. No significant changes can be observed for all chosen time points and treatments. All measurements were normalized to the control of the respective time point.

To evaluate possible molecular targets of cannabinoids in glioblastoma, the two signaling cascades FAK and p44/42 MAPK were analyzed. The protein amount and phosphorylation state were investigated at 0 min, 5 min, 10 min, 30 min, 2 h, 12 h, 24 h and 72 h after cannabinoid treatment. Thereby, no significant changes were detected. An example for p44/42 MAPK expression in U138 cells is shown in Fig 5. The exact measurement values and remaining plots are shown in supplemental S8–S10 Tables and supplemental S1–S5 Figs.

## Discussion

### Impact of cannabinoids on cell motility

Previous studies have shown that cannabinoids can alter cell motility in a receptor and cell line dependent way [17–21]. In bladder cancer cells activation of CB_2_ led to a reduced cell motility that was associated with a reduction in activity of the AKT pathway. The reduced motility was only partially reversible by the application of a CB_2_ antagonist [17]. In mammary and prostate carcinoma CB_1_ activation led to a reduced motility via the phosphorylation of FAK [18] or a reduced RhoA phosphorylation [19]. Additionally, a loss of actin filaments and a reduced cell size was reported after CB_1_ activation in prostate carcinoma cells [19]. Our results in glioblastoma cell lines differ from those obtained in other tumor entities and did not involve signaling cascades associated with FAK and p44/42 MAPK, as suggested by the literature [17–19,39]. Previous studies conducted with human and rodent glioma cells found THC, Win 55–212,2, cannabidiol and HU-210 to interact with ERK1/2 signaling [46–48,39]. Nevertheless, all these substances are neither specific for CB1 nor CB2, but target further receptors such as GPR55, TRPV1, etc. as well. Both receptors were already found to be present in the used glioblastoma cell lines [49–52]. Additionally, gliomblastoma is a highly heterogeneous tumor entity with different cell populations and cellular responses to various stimuli. Taken together, it seems possible that cannabinoid treatments result in different coupling of CB receptors and signaling cascades (reviewed in [53]). In line with this argument another aspect should be considered as the formation of cannabinoid receptor heterodimers changes coupling and signaling of these dimers upon stimulation [54–56]. Consequently, even if ERK1/2 activation is frequently modified by cannabinoids it does not seem to be a necessity in tumor cells.

Furthermore, we observed changes in the actin structure were sometimes absent, even though motility and/or cell morphology was altered, speaking against the mechanism proposed by Nithipathikom et al [19]. A reason for this discrepancy might be the systems used in previous studies, being cell lines of different origins (e.g. mammary carcinoma, prostate carcinoma) as well as the scratch wound assay that was used in most of the motility studies involving cannabinoids [17–20,22]. A further issue might be off-targets of the used cannabinoids. For example the partial agonist cannabidiol has been reported to inhibit the migration of glioblastoma cells in a cannabinoid receptor independent manner [22]. Furthermore, previous studies with cannabinoids hint to the receptors TRPV1 and GPR55 as further possible targets [57–60]. The idea of further non-CB targets of the used substances is additionally supported by the obtained data taken together with previously reported results of our group for the CB agonists JWH133 and ACEA [21]. Here the combined administration of CB agonist and antagonist was sometimes capable to cause effects relative to the control, even though the single substances did not cause significant effects (e.g. cell speed of U138 + CB2 (ant-)agonist). Furthermore, the respective antagonist was sometimes incapable of reversing the agonists effect (e.g. cell speed of LN229 + CB1 (ant-)agonist). Thus, additional off-targets of the used substances are likely. The off-targets may also explain the effect of JWH133 and AM630 being both capable to reduce the structure density in LN229 cells, but when administered together had no effect. A negative cross-talk of the downstream-signaling caused by both substances might thus be a possible explanation

Nevertheless, it remains open why the used cell lines reacted in a highly heterogeneous way to the cannabinoid treatments. An aspect that might be helpful to understand this phenomenon is the mutation status of the used cell lines. While U87 and U138 cells express wild type p53 protein, LN229 has a mutated form and PTEN is mutated in U87, not present at all in U138 and found in its wild type form in LN229 [61]. Additionally, only U87 and LN229 cells are capable to generate tumors in in vivo models [61]. These facts show the heterogeneity of the used cell lines that is also reflected by the different basal levels of the measurement parameters. PTEN, in its wild type form, is capable of regulating cell migration and motility [62]. Even though the exact mechanisms are not yet fully understood FAK and Rac1 were supposed as potential targets [62]. Similarly, mutant p53 influences a multitude of signaling cascades, mostly related to proliferation and cell cycle [63], but it also was described to enhance receptor tyrosine kinase (RTK) signaling too, including integrin recycling, epidermal growth factor receptor signaling and thus altering motility [64–67]. Consequently, if basal levels of activity or whole signaling cascades are altered it is likely that the stimulation of cannabinoid receptors might lead to fundamentally different results on the cellular level. This is supported by the known inhibitory effect of PTEN on RAC1 via Phosphatidylinositol (3,4,5)-trisphosphate (PtdIns(3,4,5)P3) and on FAK [62,68], both being potential targets of cannabinoids as well [17,18,69,70]. FAK can be directly targeted by cannabinoids, while PtdIns(3,4,5)P3 can be modulated via the activation of phosphatidylinositol-3-kinases (PI3K) [17,18,69,70]. Thus the loss of the PTEN induced inhibition of these cascades may lead to higher basal level of signaling, rendering a further cannabinoid induced activation less effective. Similarly, it was demonstrated that a cannabinoid stimulation may result in an inactivation of p53 [71],its activation [72] or having no effect on p53 [73]. All aforementioned studies were performed in different cell types and none in glioblastoma. Nevertheless, it demonstrates that cannabinoids may potentially be capable to modulate the p53 activity being another possible explanation for the diverse effects observed here.

As previously noted, we observed changes in motility, without modifications of the actin organization (U87 + AM281/AM630) and changes in actin organization without alterations in cell motility (LN229 + AM281/AM281+ACEA; U138 + AM281+ACEA/AM630+JWH133). This might be due to the fact that the lamellipodium consists mostly of dendritic actin that cannot be resolved using conventional microscopy techniques and thus changes in this network may alter motility without being optically resolved. Similarly, the composition or generated tension of the actin cortex may have changed and thus impairing or favoring cell motility or actin turnover times might be altered. Additionally, a previous study has demonstrated that U87 and C6 glioblastoma cells possess a migration mode that is independent of polymerized actin and has special requirements for Rho GTPases [74]. Consequently, the cannabinoid effects on motility in U87 cells might be mediated by alterations in the microtubule structure as well.

Under the light of the data obtained in previous studies and the data presented here it seems necessary to first elucidate whether the tumor intended for treatment is indeed sensitive to cannabinoid treatment. For some tumor entities the effect of cannabinoid stimulation was not necessarily positive [21,75], even though the majority of studies found an anti-tumoral effect in glioma [23,26,76–78]. The effect of cannabinoids was associated with the cannabinoid receptor-density, correlating lower receptor amounts with an anti-tumoral effect in astrocytoma, but this effect could not be reproduced in prostate carcinoma [79,80]. Consequently, if cannabinoids are considered to be used as a potential additional therapeutic agent its efficacy has to be evaluated for each patient separately in resected tissue.

### Association of cell motility and morphology

Another aspect of this work was to study the impact of cannabinoids on cell morphology and the relation between morphology and motility. Regarding the changes in cellular morphology and the heterogeneity of the effects, the same arguments as described for the motility can be made. Correlating the measurement parameters of live cell imaging with each other we obtained several correlations, including a correlation between cell brightness and its contact area. In a certain sense this can be regarded as a natural correlation. If we imagine a cell with a contact area *A*_*1*_ and an apparent brightness *I*_*1*_ and increase its contact area to *A*_*2*_ *> A*_*1*_ the same cell will have a lower apparent brightness *I*_*2*_ *< I*_*1*_ because it will have the same chemical composition and on average a lower height. The lower height will lead to a reduced phase shift of the transmitted light used for visualizing the cell and thus the larger cells will appear darker in phase contrast microscopy. As cell lines were used, which have a high self-similarity amongst cells of the same cell line, the given argument is highly likely. In a similar fashion the correlation between the brightness and the homogeneity of a cell can be explained. If a cell spreads out and thus decreases its apparent brightness the structure becomes less heterogeneous, even if distinct structures do not change because they have an overall lower impact due to the increased cell size. Consequently, the cell size mainly dominates the “appearance” of the cell.

A further important aspect of the live cell measurements was to investigate the relation between morphology and motility in glioblastoma. Studies of different research groups that measured both motility and morphology often did not measure both properties simultaneously in one experiment [81–84]. Thus, an association between cell shape and speed cannot clearly be made. Previous work indicated that glioblastoma cells being more polarized leads to an increase in cell motility [81–83]. Another study found the inverse correlation in glioblastoma [84]. In contrast, in our experiments we found cell speed and shape to be independent parameters. Only in U138 a moderate correlation (r = -0.44) was present indicating that an association of cell shape and speed may occur but is not a general feature in glioblastoma.

## Conclusion

In this study we could demonstrate that cannabinoids can influence cell motility, morphology and actin organization of glioblastoma cells in a cell line dependent manner but they were not mediated via signaling cascades involving p44/42 MAPK and FAK. Additionally, we have shown that morphological features, like the cell shape, are not necessarily associated with motility in glioblastoma cells.

## Supporting information

S1 Fig FAK phosphorylation of LN229 cells after cannabinoid treatment. A) depicts the phosphorylation and total amount of FAK of LN229 cells after CB_1_ agonist and inverse agonist treatment. B) shows the phosphorylation and total amount of FAK of LN229 cells after CB_2_ agonist and inverse agonist treatment. All values depict the mean of the measurements together with the sem. No significant changes can be observed for all chosen time points and treatments. All measurements were normalized to the control of the respective time point.
(TIF)

S2 Fig P44/42 MAPK phosphorylation of LN229 cells after cannabinoid treatment. A) depicts the phosphorylation of p44/42 MAPK of LN229 cells after CB_1_ agonist and inverse agonist treatment. B) shows the phosphorylation of p44/42 MAPK of LN229 cells after CB_2_ agonist and inverse agonist treatment. All values depict the mean of the measurements together with the sem. No significant changes can be observed for all chosen time points and treatments. All measurements were normalized to the control of the respective time point.
(TIF)

S3 Fig FAK phosphorylation of U87 cells after cannabinoid treatment. A) depicts the phosphorylation and total amount of FAK of U87 cells after CB_1_ agonist and inverse agonist treatment. B) shows the phosphorylation and total amount of FAK of U87 cells after CB_2_ agonist and inverse agonist treatment. All values depict the mean of the measurements together with sem. No significant changes can be observed for all chosen time points and treatments. All measurements were normalized to the control of the respective time point.
(TIF)

S4 Fig P44/42 MAPK phosphorylation of U87 cells after cannabinoid treatment. A) depicts the phosphorylation of p44/42 MAPK of U87 cells after CB_1_ agonist and inverse agonist treatment. B) shows the phosphorylation of p44/42 MAPK of U87 cells after CB_2_ agonist and inverse agonist treatment. All values depict the mean of the measurements together with the sem No significant changes can be observed for all chosen time points and treatments. All measurements were normalized to the control of the respective time point.
(TIF)

S5 Fig FAK phosphorylation of U138 cells after cannabinoid treatment. A) depicts the phosphorylation and total amount of FAK of U138 cells after CB_1_ agonist and inverse agonist treatment. B) shows the phosphorylation and total amount of FAK of U138 cells after CB_2_ agonist and inverse agonist treatment. All values depict the mean of the measurements together with the sem. No significant changes can be observed for all chosen time points and treatments. All measurements were normalized to the control of the respective time point.
(TIF)

S1 Table Results of the cell speed measurements. (DOCX)

S2 Table Results of the cell directionality measurements. (DOCX)

S3 Table Results of the contact area measurements. (DOCX)

S4 Table Results of the circularity measurements. (DOCX)

S5 Table Results of the brightness measurements. (DOCX)

S6 Table Results of the homogeneity measurements. (DOCX)

S7 Table Results of the structure density measurements. (DOCX)

S8 Table Values of the western blot analysis for U138 cells. All values are normalized to GAPDH and the control measurement of the respective time point, except for pFAK that was normalized to the total amount of FAK. The sample size is equal or larger than three.
(DOCX)

S9 Table Values of the western blot analysis for LN229 cells. All values are normalized to GAPDH and the control measurement of the respective time point, except for pFAK that was normalized to the total amount of FAK. The sample size is equal or larger than three.
(DOCX)

S10 Table Values of the western blot analysis for U87 cells. All values are normalized to GAPDH and the control measurement of the respective time point, except for pFAK that was normalized to the total amount of FAK. The sample size is equal or larger than three.
(DOCX)
