# Supplementary figures and images for: On the influence of cannabinoids on cell morphology and motility of glioblastoma cells
Source: PLoS One. 2019 Feb 12;14(2):e0212037. doi: 10.1371/journal.pone.0212037 (PMC6372232; doi:10.1371/journal.pone.0212037)

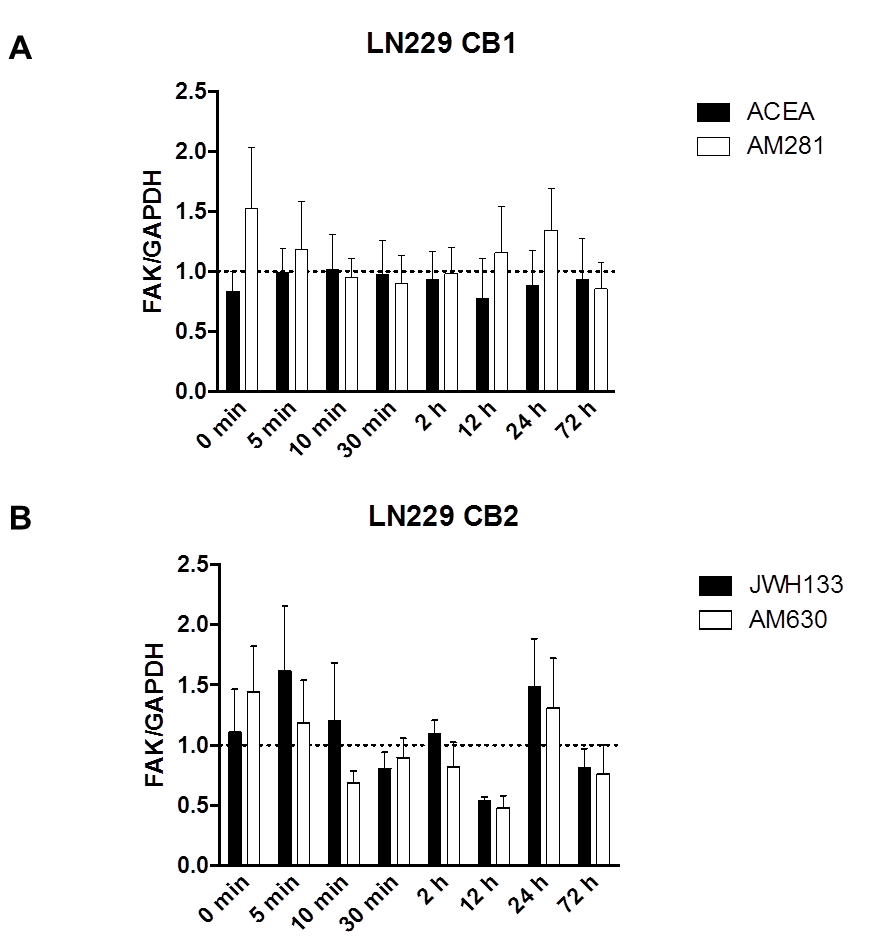

Supplement: S1 Fig — A) depicts the phosphorylation and total amount of FAK of LN229 cells after CB1 agonist and inverse agonist treatment. B) shows the phosphorylation and total amount of FAK of LN229 cells after CB2 agonist and inverse agonist treatment. All values depict the mean of the measurements together with the sem. No significant changes can be observed for all chosen time points and treatments. All measurements were normalized to the control of the respective time point. (TIF) [file pone.0212037.s001.tif]

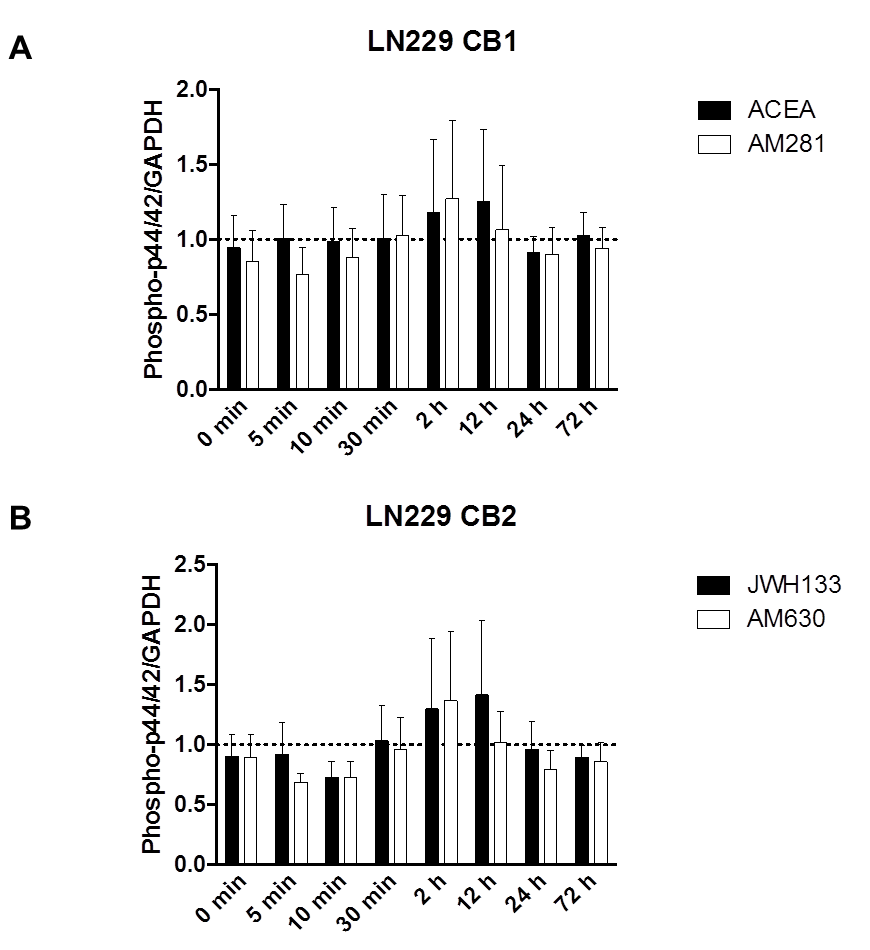

Supplement: S2 Fig — A) depicts the phosphorylation of p44/42 MAPK of LN229 cells after CB1 agonist and inverse agonist treatment. B) shows the phosphorylation of p44/42 MAPK of LN229 cells after CB2 agonist and inverse agonist treatment. All values depict the mean of the measurements together with the sem. No significant changes can be observed for all chosen time points and treatments. All measurements were normalized to the control of the respective time point. (TIF) [file pone.0212037.s002.tif]

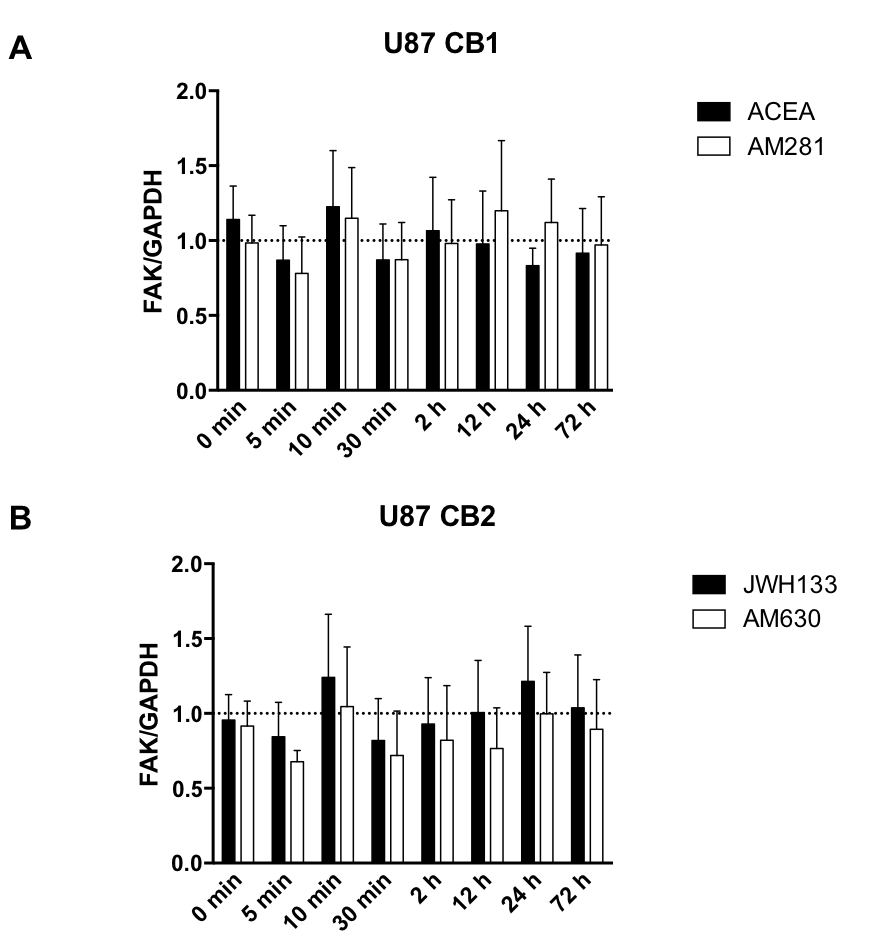

Supplement: S3 Fig — A) depicts the phosphorylation and total amount of FAK of U87 cells after CB1 agonist and inverse agonist treatment. B) shows the phosphorylation and total amount of FAK of U87 cells after CB2 agonist and inverse agonist treatment. All values depict the mean of the measurements together with sem. No significant changes can be observed for all chosen time points and treatments. All measurements were normalized to the control of the respective time point. (TIF) [file pone.0212037.s003.tif]

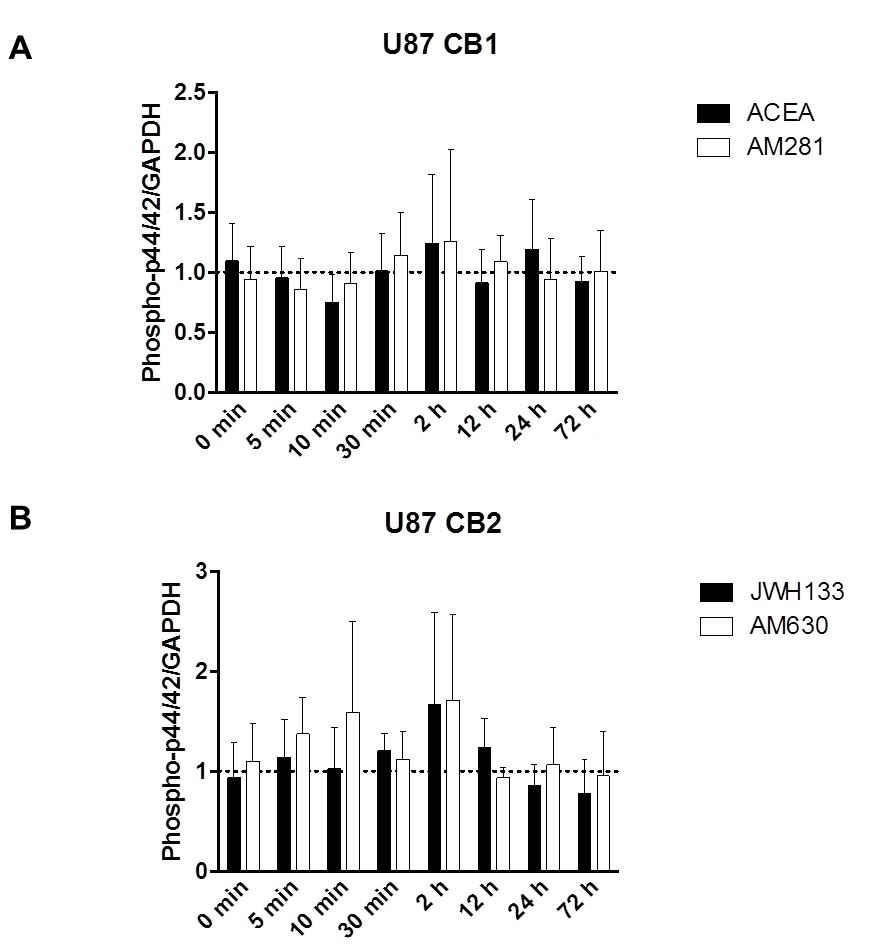

Supplement: S4 Fig — A) depicts the phosphorylation of p44/42 MAPK of U87 cells after CB1 agonist and inverse agonist treatment. B) shows the phosphorylation of p44/42 MAPK of U87 cells after CB2 agonist and inverse agonist treatment. All values depict the mean of the measurements together with the sem No significant changes can be observed for all chosen time points and treatments. All measurements were normalized to the control of the respective time point. (TIF) [file pone.0212037.s004.tif]

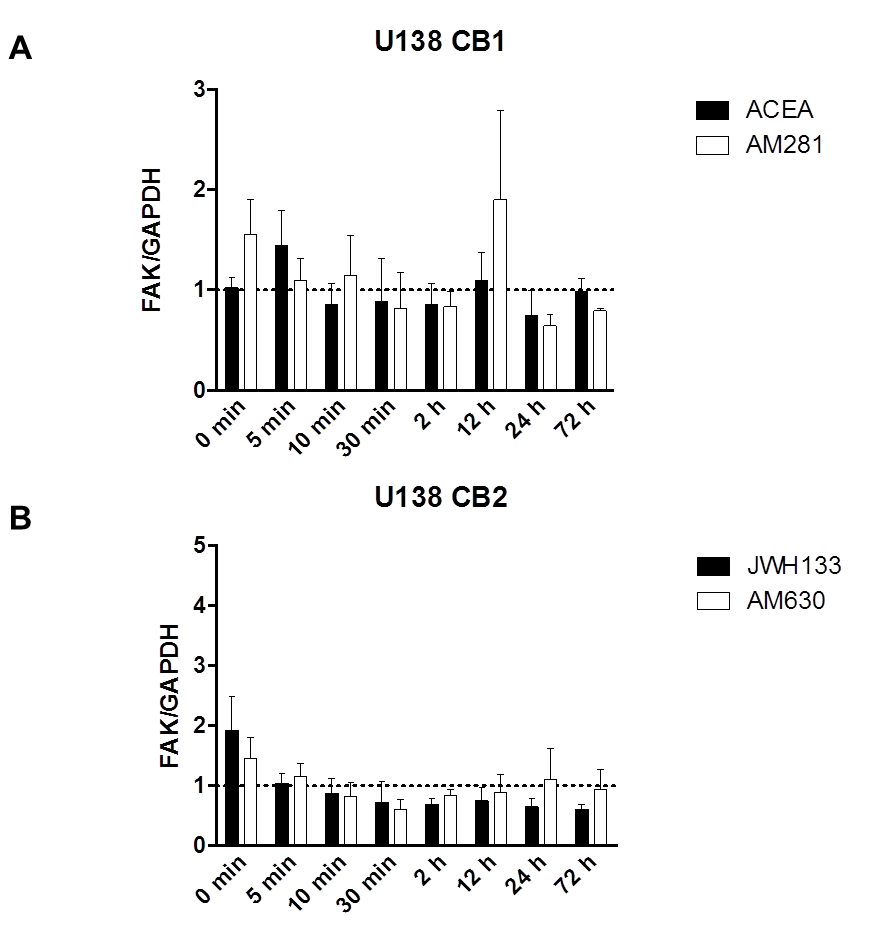

Supplement: S5 Fig — A) depicts the phosphorylation and total amount of FAK of U138 cells after CB1 agonist and inverse agonist treatment. B) shows the phosphorylation and total amount of FAK of U138 cells after CB2 agonist and inverse agonist treatment. All values depict the mean of the measurements together with the sem. No significant changes can be observed for all chosen time points and treatments. All measurements were normalized to the control of the respective time point. (TIF) [file pone.0212037.s005.tif]
