# Supplementary material for: On the influence of cannabinoids on cell morphology and motility of glioblastoma cells
Source: PLoS One. 2019 Feb 12;14(2):e0212037. doi: 10.1371/journal.pone.0212037 (PMC6372232; doi:10.1371/journal.pone.0212037)
Supplement: S1 Table — (DOCX) [file pone.0212037.s006.docx]

S1 Table. Results of the cell speed measurements.

| *Cell Type* | *Treatment* | *Mean (µm/min)* | *SEM (µm/min)* | *Sample Size* |
| --- | --- | --- | --- | --- |
| LN229 | CTL | 0.39 | 0.01 | 95 |
| LN229 | AM281 | 0.35 | 0.01 | 94 |
| LN229 | AM281+ACEA | 0.31 | 0.01 | 85 |
| LN229 | AM630 | 0.33 | 0.02 | 88 |
| LN229 | AM630+JWH133 | 0.42 | 0.02 | 120 |
| U138 | CTL | 0.34 | 0.01 | 75 |
| U138 | AM281 | 0.38 | 0.02 | 60 |
| U138 | AM281+ACEA | 0.30 | 0.01 | 74 |
| U138 | AM630 | 0.35 | 0.01 | 82 |
| U138 | AM630+JWH133 | 0.39 | 0.02 | 68 |
| U87 | CTL | 0.69 | 0.02 | 114 |
| U87 | AM281 | 0.55 | 0.05 | 53 |
| U87 | AM281+ACEA | 0.76 | 0.04 | 44 |
| U87 | AM630 | 0.59 | 0.03 | 72 |
| U87 | AM630+JWH133 | 0.76 | 0.03 | 83 |
