# Supplementary material for: On the influence of cannabinoids on cell morphology and motility of glioblastoma cells
Source: PLoS One. 2019 Feb 12;14(2):e0212037. doi: 10.1371/journal.pone.0212037 (PMC6372232; doi:10.1371/journal.pone.0212037)
Supplement: S2 Table — (DOCX) [file pone.0212037.s007.docx]

S2 Table. Results of the cell directionality measurements.

| *Cell Type* | *Treatment* | *Mean* | *SEM* | *Sample Size* |
| --- | --- | --- | --- | --- |
| LN229 | CTL | 11.6 | 1.4 | 95 |
| LN229 | AM281 | 12.7 | 3.2 | 94 |
| LN229 | AM281+ACEA | 16.8 | 5.9 | 85 |
| LN229 | AM630 | 9.2 | 1.2 | 88 |
| LN229 | AM630+JWH133 | 9.1 | 0.8 | 120 |
| U138 | CTL | 3.5 | 0.2 | 75 |
| U138 | AM281 | 18.6 | 5.8 | 60 |
| U138 | AM281+ACEA | 11.0 | 2.7 | 74 |
| U138 | AM630 | 8.2 | 1.3 | 82 |
| U138 | AM630+JWH133 | 11.4 | 4.3 | 68 |
| U87 | CTL | 7.8 | 0.6 | 114 |
| U87 | AM281 | 9.3 | 1.2 | 53 |
| U87 | AM281+ACEA | 8.4 | 1.3 | 44 |
| U87 | AM630 | 5.0 | 0.6 | 72 |
| U87 | AM630+JWH133 | 6.1 | 0.5 | 83 |
