# Supplementary material for: On the influence of cannabinoids on cell morphology and motility of glioblastoma cells
Source: PLoS One. 2019 Feb 12;14(2):e0212037. doi: 10.1371/journal.pone.0212037 (PMC6372232; doi:10.1371/journal.pone.0212037)
Supplement: S3 Table — (DOCX) [file pone.0212037.s008.docx]

S3 Table. Results of the contact area measurements.

| *Cell Type* | *Treatment* | *Mean (px)* | *SEM (px)* | *Sample Size* |
| --- | --- | --- | --- | --- |
| LN229 | CTL | 10015 | 429 | 95 |
| LN229 | AM281 | 9315 | 378 | 94 |
| LN229 | AM281+ACEA | 8410 | 390 | 85 |
| LN229 | AM630 | 9862 | 332 | 88 |
| LN229 | AM630+JWH133 | 9245 | 392 | 120 |
| U138 | CTL | 16766 | 1192 | 75 |
| U138 | AM281 | 18219 | 1381 | 60 |
| U138 | AM281+ACEA | 14550 | 763 | 74 |
| U138 | AM630 | 16404 | 1162 | 82 |
| U138 | AM630+JWH133 | 15520 | 955 | 68 |
| U87 | CTL | 7335 | 292 | 114 |
| U87 | AM281 | 8755 | 459 | 53 |
| U87 | AM281+ACEA | 7803 | 481 | 44 |
| U87 | AM630 | 8709 | 466 | 72 |
| U87 | AM630+JWH133 | 8415 | 410 | 83 |
