# Supplementary material for: On the influence of cannabinoids on cell morphology and motility of glioblastoma cells
Source: PLoS One. 2019 Feb 12;14(2):e0212037. doi: 10.1371/journal.pone.0212037 (PMC6372232; doi:10.1371/journal.pone.0212037)
Supplement: S4 Table — (DOCX) [file pone.0212037.s009.docx]

S4 Table. Results of the circularity measurements.

| *Cell Type* | *Treatment* | *Mean* | *SEM* | *Sample Size* |
| --- | --- | --- | --- | --- |
| LN229 | CTL | 0.536 | 0.007 | 95 |
| LN229 | AM281 | 0.529 | 0.008 | 94 |
| LN229 | AM281+ACEA | 0.569 | 0.008 | 85 |
| LN229 | AM630 | 0.539 | 0.008 | 88 |
| LN229 | AM630+JWH133 | 0.556 | 0.007 | 120 |
| U138 | CTL | 0.328 | 0.013 | 75 |
| U138 | AM281 | 0.334 | 0.017 | 60 |
| U138 | AM281+ACEA | 0.371 | 0.015 | 74 |
| U138 | AM630 | 0.357 | 0.011 | 82 |
| U138 | AM630+JWH133 | 0.364 | 0.011 | 68 |
| U87 | CTL | 0.453 | 0.009 | 114 |
| U87 | AM281 | 0.415 | 0.013 | 53 |
| U87 | AM281+ACEA | 0.427 | 0.011 | 44 |
| U87 | AM630 | 0.431 | 0.013 | 72 |
| U87 | AM630+JWH133 | 0.458 | 0.011 | 83 |
