# Supplementary material for: On the influence of cannabinoids on cell morphology and motility of glioblastoma cells
Source: PLoS One. 2019 Feb 12;14(2):e0212037. doi: 10.1371/journal.pone.0212037 (PMC6372232; doi:10.1371/journal.pone.0212037)
Supplement: S5 Table — (DOCX) [file pone.0212037.s010.docx]

S5 Table. Results of the brightness measurements.

| *Cell Type* | *Treatment* | *Mean* | *SEM* | *Sample Size* |
| --- | --- | --- | --- | --- |
| LN229 | CTL | 1.72 | 0.03 | 95 |
| LN229 | AM281 | 1.74 | 0.04 | 94 |
| LN229 | AM281+ACEA | 1.88 | 0.05 | 85 |
| LN229 | AM630 | 1.75 | 0.04 | 88 |
| LN229 | AM630+JWH133 | 1.78 | 0.03 | 120 |
| U138 | CTL | 1.24 | 0.03 | 75 |
| U138 | AM281 | 1.22 | 0.02 | 60 |
| U138 | AM281+ACEA | 1.38 | 0.04 | 74 |
| U138 | AM630 | 1.25 | 0.02 | 82 |
| U138 | AM630+JWH133 | 1.27 | 0.03 | 68 |
| U87 | CTL | 2.04 | 0.04 | 114 |
| U87 | AM281 | 1.95 | 0.06 | 53 |
| U87 | AM281+ACEA | 2.10 | 0.07 | 44 |
| U87 | AM630 | 1.82 | 0.05 | 72 |
| U87 | AM630+JWH133 | 1.92 | 0.05 | 83 |
