# Supplementary material for: On the influence of cannabinoids on cell morphology and motility of glioblastoma cells
Source: PLoS One. 2019 Feb 12;14(2):e0212037. doi: 10.1371/journal.pone.0212037 (PMC6372232; doi:10.1371/journal.pone.0212037)
Supplement: S6 Table — (DOCX) [file pone.0212037.s011.docx]

S6 Table. Results of the homogeneity measurements.

| *Cell Type* | *Treatment* | *Mean* | *SEM* | *Sample Size* |
| --- | --- | --- | --- | --- |
| LN229 | CTL | 29.8 | 1.0 | 95 |
| LN229 | AM281 | 27.9 | 1.1 | 94 |
| LN229 | AM281+ACEA | 31.0 | 1.2 | 85 |
| LN229 | AM630 | 30.3 | 1.3 | 88 |
| LN229 | AM630+JWH133 | 31.2 | 1.0 | 120 |
| U138 | CTL | 18.9 | 1.0 | 75 |
| U138 | AM281 | 16.9 | 1.1 | 60 |
| U138 | AM281+ACEA | 21.+ | 1.1 | 74 |
| U138 | AM630 | 17.8 | 0.9 | 82 |
| U138 | AM630+JWH133 | 18.2 | 1.2 | 68 |
| U87 | CTL | 39.3 | 1.3 | 114 |
| U87 | AM281 | 36.5 | 2.3 | 53 |
| U87 | AM281+ACEA | 41.4 | 3.5 | 44 |
| U87 | AM630 | 32.1 | 2.3 | 72 |
| U87 | AM630+JWH133 | 35.1 | 1.6 | 83 |
