# Supplementary material for: On the influence of cannabinoids on cell morphology and motility of glioblastoma cells
Source: PLoS One. 2019 Feb 12;14(2):e0212037. doi: 10.1371/journal.pone.0212037 (PMC6372232; doi:10.1371/journal.pone.0212037)
Supplement: S7 Table — (DOCX) [file pone.0212037.s012.docx]

S7 Table. Results of the structure density measurements.

| *Cell Type* | *Treatment* | *Mean* | *SEM* | *Sample Size* |
| --- | --- | --- | --- | --- |
| LN229 | CTL | 0.346 | 0.006 | 84 |
| LN229 | ACEA | 0.308 | 0.005 | 83 |
| LN229 | AM281 | 0.296 | 0.010 | 84 |
| LN229 | AM281+ACEA | 0.352 | 0.008 | 76 |
| LN229 | JWH133 | 0.282 | 0.006 | 78 |
| LN229 | AM630 | 0.319 | 0.005 | 77 |
| LN229 | AM630+JWH133 | 0.356 | 0.012 | 17 |
| U138 | CTL | 0.335 | 0.006 | 83 |
| U138 | ACEA | 0.356 | 0.008 | 83 |
| U138 | AM281 | 0.352 | 0.008 | 83 |
| U138 | AM281+ACEA | 0.342 | 0.007 | 87 |
| U138 | JWH133 | 0.339 | 0.007 | 81 |
| U138 | AM630 | 0.352 | 0.009 | 65 |
| U138 | AM630+JWH133 | 0.345 | 0.010 | 30 |
| U87 | CTL | 0.321 | 0.006 | 94 |
| U87 | ACEA | 0.309 | 0.006 | 82 |
| U87 | AM281 | 0.316 | 0.006 | 84 |
| U87 | AM281+ACEA | 0.335 | 0.008 | 72 |
| U87 | JWH133 | 0.310 | 0.008 | 84 |
| U87 | AM630 | 0.314 | 0.007 | 83 |
| U87 | AM630+JWH133 | 0.336 | 0.006 | 105 |
