# Supplementary material for: On the influence of cannabinoids on cell morphology and motility of glioblastoma cells
Source: PLoS One. 2019 Feb 12;14(2):e0212037. doi: 10.1371/journal.pone.0212037 (PMC6372232; doi:10.1371/journal.pone.0212037)
Supplement: S8 Table — All values are normalized to GAPDH and the control measurement of the respective time point, except for pFAK that was normalized to the total amount of FAK. The sample size is equal or larger than three. (DOCX) [file pone.0212037.s013.docx]

S8 Table. Values of the Western Blot analysis for U138 cells. All values are normalized to GAPDH and the control measurement of the respective time point, except for pFAK that was normalized to the total amount of FAK. The sample size is equal or larger than three.

| **Time** | **Treatment** | **Phospho-p44/42 MAPK** | | **FAK** | | **pFAK/FAK** | |
| --- | --- | --- | --- | --- | --- | --- | --- |
| **U138** |  | **Mean** | **SEM** | **Mean** | **SEM** | **Mean** | **SEM** |
| 0 min | CTL | 1.000 | 0.449 | 1.000 | 0.282 | 1.000 | 0.194 |
|  | ACEA | 1.026 | 0.412 | 1.022 | 0.100 | 0.855 | 0.161 |
|  | AM281 | 0.910 | 0.397 | 1.555 | 0.349 | 1.238 | 0.329 |
|  | JWH133 | 1.046 | 0.386 | 1.925 | 0.563 | 1.336 | 0.397 |
|  | AM630 | 1.027 | 0.245 | 1.447 | 0.357 | 0.908 | 0.198 |
| 5 min | CTL | 1.000 | 0.285 | 1.000 | 0.176 | 1.000 | 0.212 |
|  | ACEA | 0.954 | 0.037 | 1.445 | 0.348 | 1.117 | 0.297 |
|  | AM281 | 1.133 | 0.108 | 1.093 | 0.217 | 0.735 | 0.132 |
|  | JWH133 | 1.336 | 0.147 | 1.041 | 0.167 | 0.912 | 0.261 |
|  | AM630 | 1.222 | 0.138 | 1.157 | 0.209 | 0.846 | 0.191 |
| 10 min | CTL | 1.000 | 0.243 | 1.000 | 0.190 | 1.000 | 0.329 |
|  | ACEA | 0.765 | 0.042 | 0.851 | 0.214 | 0.790 | 0.230 |
|  | AM281 | 0.952 | 0.209 | 1.148 | 0.397 | 1.023 | 0.343 |
|  | JWH133 | 0.943 | 0.214 | 0.875 | 0.250 | 1.178 | 0.103 |
|  | AM630 | 0.935 | 0.112 | 0.826 | 0.228 | 0.809 | 0.150 |
| 30 min | CTL | 1.000 | 0.192 | 1.000 | 0.442 | 1.000 | 0.489 |
|  | ACEA | 1.130 | 0.388 | 0.889 | 0.425 | 0.955 | 0.487 |
|  | AM281 | 0.999 | 0.455 | 0.815 | 0.362 | 1.231 | 0.742 |
|  | JWH133 | 1.005 | 0.342 | 0.729 | 0.337 | 0.914 | 0.449 |
|  | AM630 | 0.945 | 0.408 | 0.612 | 0.164 | 0.694 | 0.260 |
| 2 h | CTL | 1.000 | 0.413 | 1.000 | 0.278 | 1.000 | 0.354 |
|  | ACEA | 0.764 | 0.195 | 0.851 | 0.215 | 0.757 | 0.218 |
|  | AM281 | 0.813 | 0.207 | 0.835 | 0.154 | 0.885 | 0.261 |
|  | JWH133 | 0.768 | 0.124 | 0.689 | 0.096 | 0.615 | 0.143 |
|  | AM630 | 1.578 | 0.366 | 0.845 | 0.093 | 1.000 | 0.119 |
| 12 h | CTL | 1.000 | 0.208 | 1.000 | 0.228 | 0.815 | 0.185 |
|  | ACEA | 1.361 | 0.545 | 1.097 | 0.273 | 0.639 | 0.310 |
|  | AM281 | 1.672 | 0.877 | 1.901 | 1.258 | 1.276 | 0.954 |
|  | JWH133 | 1.785 | 0.844 | 0.751 | 0.220 | 0.397 | 0.160 |
|  | AM630 | 1.864 | 0.729 | 0.889 | 0.306 | 0.425 | 0.216 |
| 24 h | CTL | 1.000 | 0.272 | 1.000 | 0.320 | 1.000 | 0.421 |
|  | ACEA | 1.402 | 0.574 | 0.747 | 0.251 | 0.854 | 0.417 |
|  | AM281 | 0.989 | 0.173 | 0.640 | 0.110 | 0.676 | 0.321 |
|  | JWH133 | 0.920 | 0.273 | 0.655 | 0.132 | 0.608 | 0.289 |
|  | AM630 | 0.838 | 0.220 | 1.112 | 0.506 | 0.958 | 0.595 |
| 72 h | CTL | 1.000 | 0.217 | 1.000 | 0.151 | 1.000 | 0.590 |
|  | ACEA | 1.088 | 0.140 | 0.987 | 0.129 | 0.734 | 0.410 |
|  | AM281 | 1.124 | 0.177 | 0.791 | 0.026 | 0.522 | 0.256 |
|  | JWH133 | 0.858 | 0.197 | 0.614 | 0.078 | 0.415 | 0.098 |
|  | AM630 | 0.786 | 0.181 | 0.935 | 0.344 | 0.698 | 0.468 |
